# Multifunctional Indium Tin Oxide Electrode Generated by Unusual Surface Modification

**DOI:** 10.1038/srep36708

**Published:** 2016-11-18

**Authors:** Sarra Bouden, Antoine Dahi, Fanny Hauquier, Hyacinthe Randriamahazaka, Jalal Ghilane

**Affiliations:** 1 Nano-Electro-Chemistry group, Université Paris Diderot, Sorbonne Paris Cité, ITODYS, UMR 7086 CNRS, 15 rue Jean-Antoine de Baïf, 75205 Paris, France; 2 LICSEN, NIMBE, CEA, CNRS, Université Paris-Saclay, CEA Saclay 91191 Gif-sur-Yvette Cedex, France - CNAM, Department of Chemistry and Health & Life Sciences, 292 Rue Saint-Martin, 75003 Paris, France

## Abstract

The indium tin oxide (ITO) material has been widely used in various scientific fields and has been successfully implemented in several devices. Herein, the electrochemical reduction of ITO electrode in an organic electrolytic solution containing alkali metal, NaI, or redox molecule, N-(ferrocenylmethyl) imidazolium iodide, was investigated. The reduced ITO surfaces were investigated by X-ray photoelectron spectroscopy and grazing incident XRD demonstrating the presence of the electrolyte cation inside the material. Reversibility of this process after re-oxidation was evidenced by XPS. Using a redox molecule based ionic liquid as supporting electrolyte leads to fellow electrochemically the intercalation process. As a result, modified ITO containing ferrocenyl imidazolium was easily generated. This reduction process occurs at mild reducing potential around −1.8 V and causes for higher reducing potential a drastic morphological change accompanied with a decrease of the electrode conductivity at the macroscopic scale. Finally, the self-reducing power of the reduced ITO phase was used to initiate the spontaneous reduction of silver ions leading to the growth of Ag nanoparticles. As a result, transparent and multifunctional active ITO surfaces were generated bearing redox active molecules inside the material and Ag nanoparticles onto the surface.

Indium tin oxide (ITO) has been widely used as electrode material in various scientific fields. ITO films exhibit high conductivity and high optical transparency. Due to these characteristics, ITO films are successfully implemented in several devices such as electrochromism^1^,^2^, photovoltaics^3^,^4^, sensors^5^, plasmonic devices^6^,^7^, and light emitting diodes^8^,^9^. The common purpose of these applications is based on the use of modified ITO surface. To this end, numerous strategies were reported in the literature including self-assembled monolayers^10^,^11^, spin coating^12^, electrochemical polymerization^13^, and electrochemical grafting through the reduction of diazonium derivatives^14^. These surfaces modifications methods generate a new interface with specific properties that could be easily tuned leading to their integration in various devices. Most of the applications described above are based on the investigation of electron transfer process at the interface. Thus, understanding the ITO interface is an important issue. In this context, the cathodic and anodic corrosion of ITO electrode has been reported^15^,^16^,^17^,^18^,^19^. For example in acidic media, the cathodic polarization of ITO conduces to the formation of metallic particles (In-Sn) onto the surface^15^,^16^. The interaction between alkali metal and ITO material has been reported^20^,^21^. Aliev *et al*. reported the reversible superconductivity in electrochemically intercalated indium-tin oxide films^22^,^23^. They investigate the effect of the electrochemical doping of ITO films with alkali metals, using anodic charging process, on the paramagnetic Meissner effect (PME).

Another electrochemical process, less investigated and conduces to surface modification, is the electrochemical reduction of electrode materials in dry electrolytic solution. This phenomenon is observed during the basic electrochemical process when performing the so-called “electrochemical window or background electrolyte”. Contrarily to the classical surface modification, this electrochemical process is accompanied by the insertion of cation inside the materials. During the cathodic polarization of the electrode material, in dry electrolytic solution containing alkali metals or quaternary ammonium salts, inclusion of the electrolyte cation into the material occurs generating “solid phase”. This transformation has been reported in the case of carbon, gold and platinum electrodes^24^,^25^,^26^,^27^,^28^,^29^. The occurrence of the metallic phase transition has been evidenced using different methods, including scanning electrochemical microscopy (SECM), electrochemical atomic force microscopy (EC-AFM), and X-ray photoelectron spectroscopy (XPS)^30^,^31^,^32^,^33^. More recently, the possibility to generate this new phase in the presence of tasks specific ionic liquid was reported^34^,^35^,^36^. Moreover, the reducing power of the generated material was successfully employed to initiate a self-reduction process, generating a bi-functional redox system^29^,^30^,^37^,^38^. However, this process remains a relatively less developed and was not investigated on semiconductor material such as ITO films.

In this work, we purpose to investigate the electrochemical reduction of ITO electrode in dry organic solvent. The cathodic polarization of ITO in acetonitrile solution containing sodium iodide as electrolyte is performed. The change in the ITO surface upon the cathodic treatment was investigated by X-ray photoelectron spectroscopy (XPS), atomic force microscopy (AFM) and X-ray diffraction (XRD). Besides that, the cathodic activation of ITO was performed in the presence of 1-ferrocenylmethyl-3-methylimidazolium iodide (FcMImI), as supporting electrolyte. In this case, the characterization was achieved using XPS and cyclic voltammetry. Finally, the reduced ITO film was engaged in self-reductive process in the presence of silver ions. The resulting modified surface was characterized by XPS, electrochemistry, scanning electron microscope and surface plasmon spectroscopy. Our purpose in this work was to answer to several fundamental questions related to the electrochemical reduction of ITO in organic electrolytic solution, electrochemical reversibility of the process, the surface and chemical composition changes, and the use of the reduced material to induce self-reduction process.

## Results and Discussion

**Figure 1:**
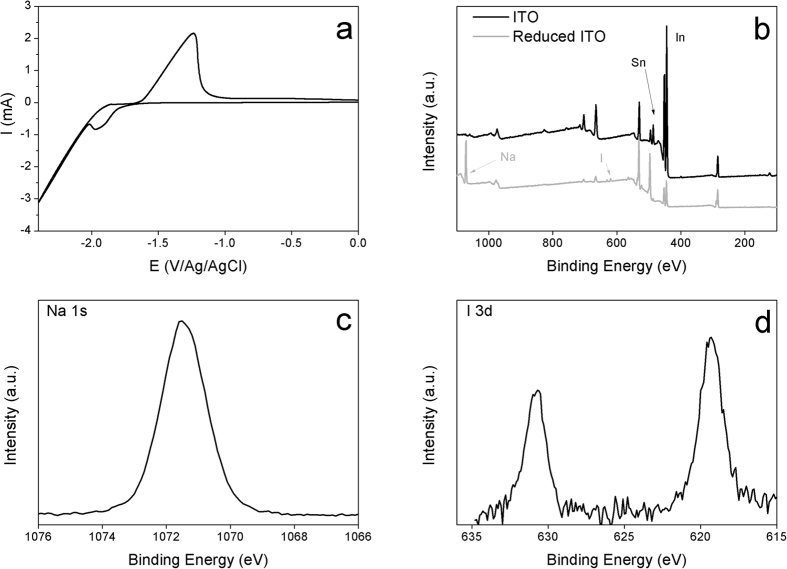
(**a**) Cyclic voltammogram recorded on ITO electrode in ACN containing 0.1 M NaI. Scan rate 0.1 V.s^−1^. (**b**) XPS survey scans of untreated ITO (black line) and reduced ITO at −2 V in ACN containing 0.1 M NaI during 100 s (gray line). (**c**,**d**) XPS high resolution spectra for Na(1s) and I(3d).

The cyclic voltammetry recorded on ITO electrode in acetonitrile (ACN) solution containing 0.1 M NaI displays a quasi-reversible redox system with a reduction peak potential at −2 V (Fig. 1a). In comparison with the reported results on Pt^38^, the observed reduction peak could be ascribed to the reduction of ITO concomitant with the insertion of Na^+^ into the electrode material. Following that, cathodic polarization of the ITO sample at −2 V during 100 s was performed. Then, the sample was analyzed by XPS measurements after being rinsed and sonicated in ACN (Fig. 1).

The comparison of the survey spectra, before and after cathodic polarization of ITO, reveals several features. After the cathodic treatment the XPS survey shows a decrease in the intensity of In(3d_5/2_) and Sn(3d_5/2_) peaks reflecting the presence of surface modification. Besides that, new peaks appear at 1071.5 and 619.1 eV corresponding to Na(1s) and I(3d_5/2_), respectively. A deeper examination of XPS data shows a background increase in the survey spectrum after the Na(1s) peak indicating the inelastic scattering of the photoelectrons. Similar tendencies are observed for O(1s) (peak at 532.0 eV), In(3d_5/2_) and Sn(3d_5/2_), those elements are located onto and into the ITO surface. This behavior is not observed for C(1s) (peak at 285.0 eV) and I(3d) suggesting that the sodium is deeply located inside the substrate. This first result confirms that the cathodic treatment of ITO induces surface chemical composition change and highlights the incorporation of Na^+^ into the ITO sample in similar way as reported for Pt electrode^33^. This study was complemented by performing XPS investigation of various ITO samples after cathodic activation at −2 V using different injection charge density. The obtained results are summarized in Table 1. One has to note that the given atomic percentages values reflect only a tendency in the composition variation occurring near the surface.

Overall, increasing the charge density conduces to a decrease of the atomic percentage of In and Sn, while the atomic percentage of Na increases and the iodine remain relatively constant. The atomic percentage of iodine is found to be very low when compared to Na and appears to be less sensitive to the increase of the injected charge suggesting that its presence is mainly due to surface adsorbed species. In contrast, the Na atomic percentage is affected by the injected charge and reaches a constant value after the injection of a charge around −2.4 C/cm^2^. This saturation could suggest a decrease of the intercalation rate. These experiments demonstrate the progressive Na^+^ intercalation into ITO electrode with the injected cathodic charge.

**Figure 2:**
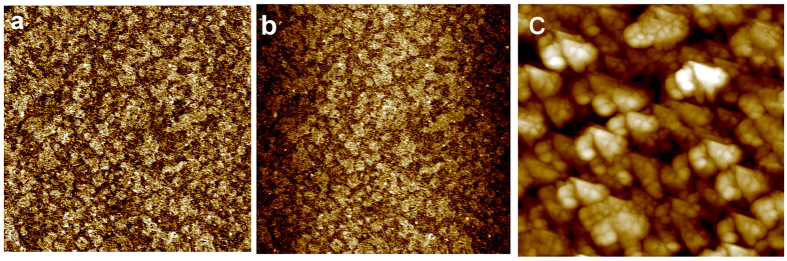
AFM images of ITO electrode (**a**) bare ITO. (**b**,**c**) after electrochemical polarization during 100 s in acetonitrile solution containing 0.1 M NaI at −1 V at −2 V, respectively. Image size 5 × 5 μm^2^.

Furthermore, the morphological change of the ITO sample after the polarization was investigated using atomic force microscopy. Figure 2 compares the AFM images recorded before (Fig. 2a) and after performing the cathodic treatment in acetonitrile solution containing 0.1 M NaI at −1 V (Fig. 2b) and −2 V (Fig. 2c) during 100 s.

**Figure 3:**
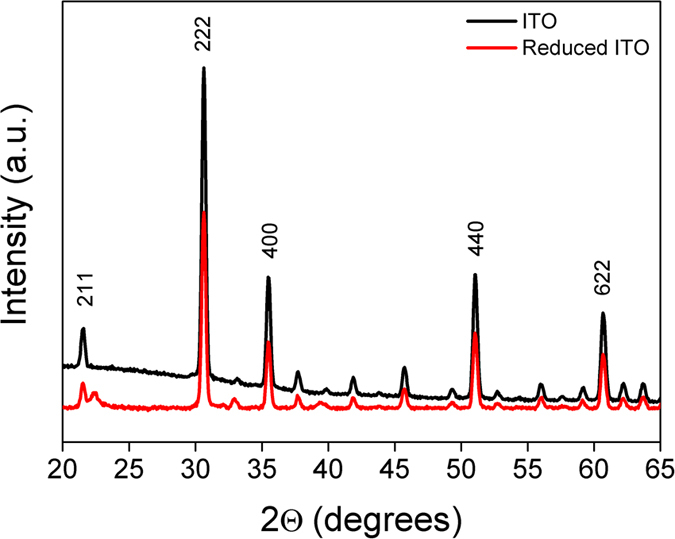
Grazing incident X-ray diffraction of bare ITO (blak line) and reduced ITO at −2 V in the presence of NaI (red curve).

The AFM image on bare ITO, Fig. 2a, shows homogeneous and smooth surface with an average roughness of about 1.5 nm. After a cathodic polarization at a boundary potential more positive than −1 V, the AFM image in Fig. 2b shows similar morphology as observed for bare ITO confirming the absence of any change on the surface morphology and roughness at these potentials. However, after polarization at −2 V the initial morphology of the surface was completely changed and been replaced by large spheroids with a diameter of about 300 nm (Fig. 2c). In addition, the morphological change of the ITO surface is accompanied by the increase of the average surface roughness to a value of 160 nm. The average roughness increased to reach a value 100 times higher than the initial roughness obtained on bare ITO. One has to note, that the initial ITO film thickness is around 100 nm. Consequently, after the ITO reduction the notable morphological change accompanied with roughness increases suggests the change in the film thickness and the occurrence of surface swelling. This surface swelling is associated to the insertion of Na^+^ into ITO electrode inducing a volume change and the expansion of the electrode material. The electrochemically activated ITO film was further investigated using grazing incident X-ray diffraction GI-XRD (Fig. 3).

The GI-XRD patterns recorded for bare ITO sample (black line in Fig. 3) revealed five orientation peaks observed along (211), (222), (400), (440) and (622) directions characteristic of a cubic phase ITO^39^,^40^. After polarization at −2 V, the GI-XRD pattern (red line in Fig. 3) shows similar 2θ lines as observed for bare ITO. In addition to these lines, the electrochemically reduced ITO exhibited additional peaks at 22.3°, 33° and 39°. The appearance of these new peaks suggests strongly the structural change of the ITO material upon the electrochemical treatment. These peaks could be considered as a signature of the formation of new phase between incorporated sodium and ITO film. In addition, the obtained X-ray diffraction patterns shows peaks characteristic of orthorhombic structure with lattice parameters (a = 8.7 A°, b = 7.9 A°, c = 4.1 A°).

**Figure 4:**
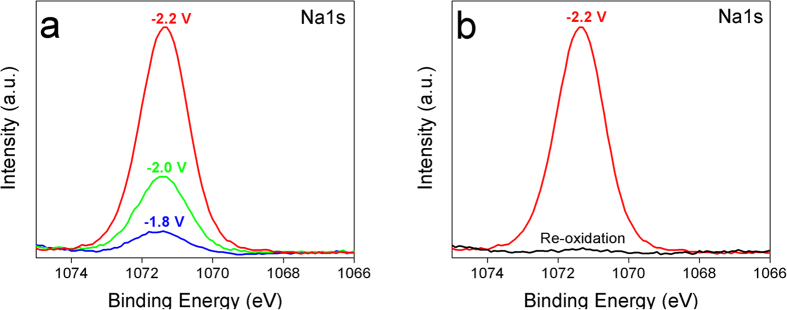
XPS high resolution spectra for Na(1s) (**a**) reduced ITO in acetonitrile containing 0.1 M NaI at different reducing potential −2.2 V (red line) −2 V (green line) −1.8 V (blue line), polarization time 50 s. (**b**) Compare the XPs spectrum of Na(1s) recorded on reduced ITO at −2.2 V (red line) and after re-oxidation of the same sample at 0.5 V (black line).

To bring more insight concerning the occurrence of the intercalation of the Na ions into the ITO film, during the electrochemical reduction, the reversibility of the process was investigated. First, the ITO reduction was performed at different reducing potentials (−1.8, −2 and −2.2 V) during 50 s and the XPS spectrum of Na(1s) element was recorded (see Fig. 4a).

The XPS spectra show clearly the increase of the amount of Na within the ITO film with the increase of the reducing potential. The atomic percentage of Na^+^ increases from 2% at −1.8 V to 5% at −2 V and reaches 14% at −2.2 V. However, the atomic percentage of the anion, I^−^, remains relatively stable around 0.5% and appears to be independent of the applied potential during the reduction process. This result indicates clearly that the observed amount of I^−^ is mainly due to surface adsorbed species. In contrarily, applying more negative potential induces the increase of the atomic percentage of Na element suggesting the incorporation of more Na into ITO film. Finally, the ITO sample polarized at −2.2 V was re-oxidized in the same solution by applying anodic potential at 0.5 V during 50 s. Figure 4b shows the narrow XPS spectra of Na(1s) obtained on reduced ITO (red line) and on re-oxidized ITO (black line). The figure shows clearly the absence of Na(1s) signal after re-oxidation confirming the electrochemical reversibility of the process. The obtained results could not be basically explained by the electrolyte adsorption or the formation of the solid electrolyte interphase (SEI) on the ITO film. Overall, all the results described above suggest strongly that the reduction of ITO electrode in the presence of NaI induces Na intercalation within the ITO material.

**Figure 5:**
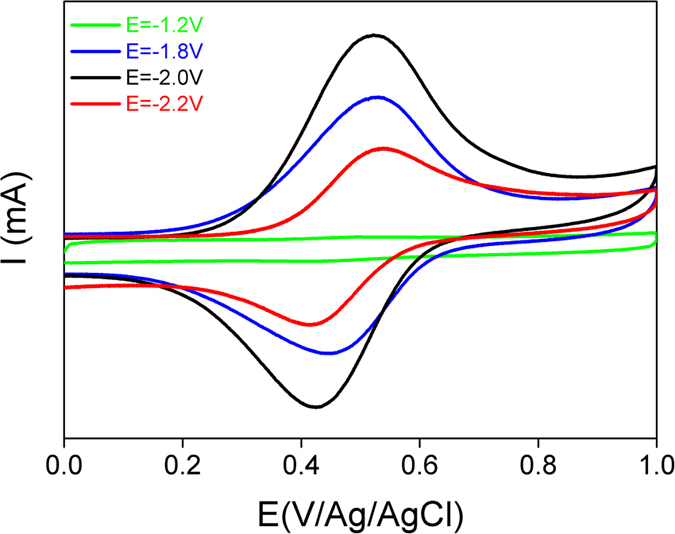
CV’s of ITO electrodes in acetonitrile containing 0.1 M Bu_4_NBF_4_, after cathodic polarization at different potentials during 100 s. Scan rate 0.1 V/s. Electrode area 1 cm^2^.

In this part, the electrochemical activation of ITO was investigated in acetonitrile solution containing redox supporting electrolyte. Thus, 1-ferrocenylmethyl-3-methylimidazolium iodide (FcMImI) was synthesized (see Supporting Information SI) and was used as supporting electrolyte. The use of redox active electrolyte allows the electrochemical characterization of reduced ITO by targeting the redox signal of ferrocenyl unit. The cathodic polarization, during 100 s, of various ITO samples was performed in ACN solution containing 0.1 M (FcMImI) at different applied potentials (i.e. −1.2, −1.8, −2 and −2.2 V). Next, the samples were thoroughly rinsed and sonicated in ACN and then immersed in acetonitrile solution containing 0.1 M Bu_4_NBF_4_. The electrochemical CV’s responses of the different ITO electrodes are summarized in Fig. 5.

**Figure 6:**
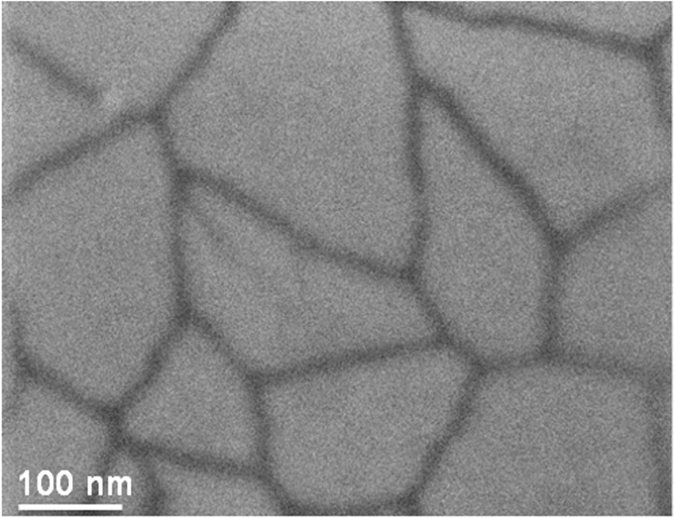
SEM image of ITO after electrochemical reduction in acetonitrile solution containing 0.1 M FcImI at −2.6 V during 100 s.

For polarized ITO electrode at a potential higher than −1.2 V no electrochemical signal of Fc is observed (green curve) indicating the absence of surface modification. However, after polarization between −1.8 and −2.2 V the characterizations exhibit a reversible redox signal at 0.5 V corresponding to ferrocenium/ferrocene imidazolium redox couple. Moreover, the analysis of the electrochemical signal on Fig. 5 could provide estimation of the amount of the redox active molecules using the formula *Γ* = *Q/nFS*. Where *Q* corresponds to the charge measured by integration of the anodic peak, *n* is the number of the electron, *F* is the faraday constant and *S* is the area of the electrode. In the present case, the average surface concentration, after ITO reduction at −2 V, was found around 3.5 × 10^−9^ mol × cm^−2^. The comparison of the different CV’s shows an increase of the anodic peak current for the ITO sample polarized at −1.8 and −2 V, respectively. Nevertheless, after polarization at −2.2 V the peak intensity decreases. These experiments have been repeated several times and show similar behavior. In order to understand the origin of the decrease of the Fc electroactivity, the ITO reduction was performed at higher reducing potential −2.4 and −2.6 V. In both cases, the electrochemical characterization of the ITO sample in electrolytic solution shows the absence of Fc redox signal (as shown in Fig. S2a,b) and resistive behavior is observed suggesting the change in the conductivity of the ITO electrode. This sample was characterized by SEM image as presented in Fig. 6.

The SEM image exhibits a drastic morphological change of the substrate and shows clearly the formation of craters and the presence of isolated ITO islands with a size ranged from 100 to 200 nm separated by 20 nm distances. In this case, the absence of the Fc signal, after cathodic polarization at E ≤ −2.4 V, is mainly due to the change in the conductivity of the ITO from macroscopic to microscopic scale. One has to note that the used electrode is based on 100 nm thick ITO film deposited onto glass, and thus the observed craters suggest that a deeper intercalation of the supporting electrolyte cation into the ITO is occurring. Upon applying more negative potential, <−2.6 V, the ITO surface turns dark causing a loss of its transparency and conductivity. The sheet resistance of the reduced ITO films was measured using a standard four-point probe technique. As a result, the sheet resistance of the as received ITO is around 10 Ω/sq, while after the cathodic treatment at −2.6 V the sheet resistance increased by 3 order of magnitude confirming the change of ITO conductivity at higher cathodic potential.

**Figure 7:**
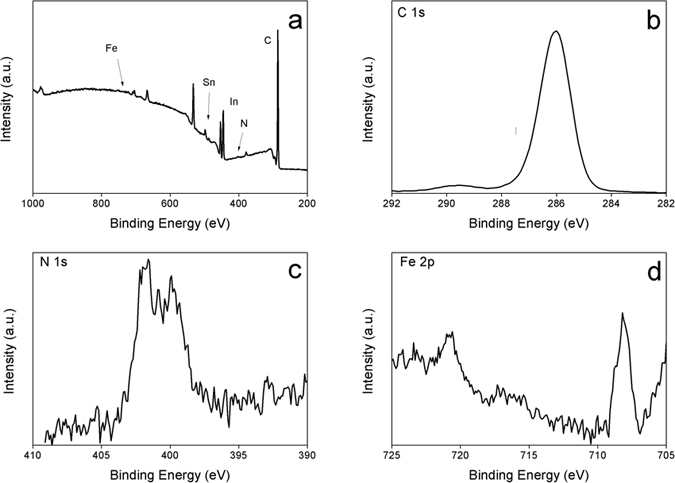
XPS survey scan of reduced ITO at −2 V during 100 s in ACN containing 0.1 M FcMImI, and XPS high resolution spectra of C(1s), N(1s) and Fe(2p).

Overall, the cathodic polarization between −1.8 and −2.4 V induces intercalation of FcMIm cation into ITO electrode and for more negative polarization morphological and conductivity changes are observed. The modified ITO sample at −2 V during 100 s was further characterized by XPS as shown in Fig. 7.

Compared to unmodified ITO sample (Fig. 1b gray line) the survey XPS spectrum shows a strong attenuation of the In(3d) and Sn(3d) signal while the C(1s) signal increases. Besides that, additional peaks corresponding to N(1s) and Fe(2p) components were observed. The Fe(2p) high resolution spectrum evidences the presence of two peaks at 708.2 and 720.8 eV attributed to the Fe(2p_3/2_) and Fe(2p_1/2_), respectively. The presence of these peaks at these binding energies values is in agreement with those previously reported for immobilized ferrocenyl moieties onto electrode surface and corresponds to ferrocenyl in its reduced state^41^. The N(1s) spectrum shows two components at 400 and 401.7 eV attributed to reduced nitrogen (from surface contamination) and to imine group in the form of imidazolic ring, respectively^41^. In addition, the C(1s) signal is composed of three partially resolved peaks at 285, 286.7 and 288.1 eV. The peak at low binding energy is contribution of aromatic carbon while the other peaks correspond to C—N band in the imidazolium ring^42^. Moreover, the background increases after the C(1s) and Fe(2p) signals are a signature of the presence of these elements inside the ITO material. Overall, the XPS and the electrochemical results evidence the presence and the intercalation of ferrocenyl-imidazolium moieties on ITO and suggest the absence of the formation of solid electrolyte interphase (SEI).

Furthermore, the electrochemical reduction in the presence of FcMImI as supporting electrolyte was performed using similar experimental conditions as described above but with fluorine doped tin oxide (FTO) as electrode. In this case, after cathodic polarization of FTO electrode at −2.2 V, the electrochemical characterizations does not shows the presence of electroactive signal corresponding to ferrocenyl redox group, and the XPS investigations of the reduced FTO, at different potentials, confirm the absence of Fe(2p) signal at the FTO surface. These results reveal several features: (i) the intercalation of the supporting electrolyte cation occurs with ITO and that the same phenomenon is not observed with FTO material, (ii) the possibility of the formation of solid electrolyte interphase (SEI) as explanation for surface modification could be excluded since the latter should not depend on the nature of the electrode material (iii) for ITO material the electrolyte cation intercalation occurs specially within the indium oxide (In_2_O_3_).

**Figure 8:**
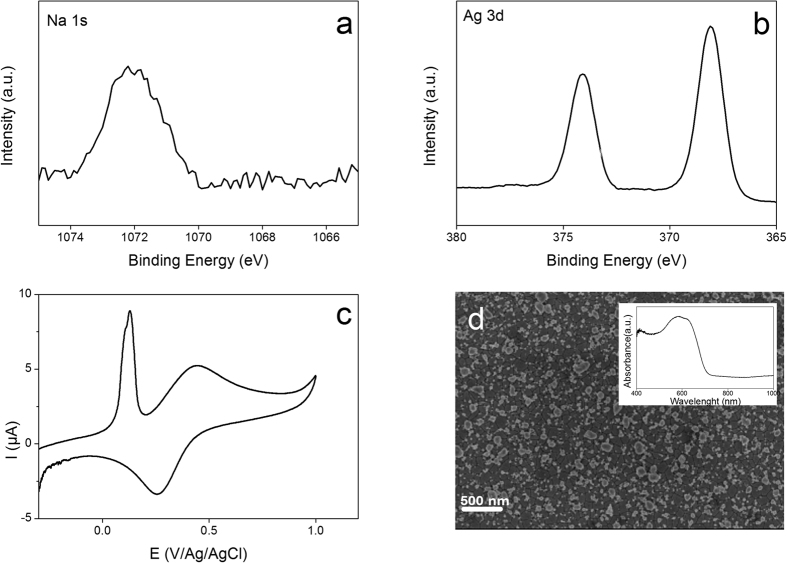
(**a**,**b**) XPS high resolution spectra of Na(1s) and Ag(3d) element recorded on reduced ITO sample, in 0.1 M NaI at −2 V during 100 s, followed by 10 min immersion in 0.01 M AgClO_4_ aqueous solution. (**c**,**d**) represent the CV in aqueous 0.1 M KCl solution and SEM image of reduced ITO at −2.2 V, in 0.1 M FcMImI during 100 s, followed by immersion in 0.01 M AgClO_4_. The inset in Fig. 6d represents the optical extinction spectrum.

As reported in the introduction, the reactivity of reduced platinum was successfully employed to induce the reduction of diazonium salts^34^,^37^. In this part, after reducing the ITO at −2 V during 100 s in the presence of 0.1 M NaI or FcMImI, the sample was immersed in aqueous solution containing 0.01 M AgClO_4_ during 10 min without the assistance of external potential. Next, the modified ITO samples were characterized by XPS (case of NaI, see Fig. 8a,b), while for the ITO reduction in the presence of FcMImI, the resulting materials were investigated using cyclic voltammetry and SEM image (Fig. 8c,d).

Figure 8a shows the presence of peak corresponding to Na(1s) highlighting the incorporation of Na cation after the cathodic polarization and reaction with Ag^+^ ions. Besides that, two new peaks appear at 368 and 374.3 eV attributed to the Ag(3d_5/2_) and Ag(3d_3/2_) signals, respectively (Fig. 8b). Deeper analysis of Ag signal leads to determine the Auger parameter (α) which is defined as the sum of the kinetic energy of the Auger electron and the XPS binding energy of Ag(3d_5/2_)^43^. This parameter was generally used to determine the oxidation state of metal. The calculated Auger parameter is around 726 eV confirming the presence of metallic Ag rather than silver oxide^44^. This result confirms the spontaneous reduction of Ag^+^ to Ag onto reduced ITO while the Na^+^ remains inside the material. In the case of redox electrolyte, the electrochemical characterization in aqueous KCl solution (Fig. 8c) shows clearly the presence of sharp oxidation peak at 0.11 V corresponding to Ag striping. Besides that, a redox reversible system at 0.34 V attributed to the electroactivity of the incorporated FcMIm cation is observed. This experiment evidences the formation of multifunctional ITO surface containing redox molecules into the ITO and Ag onto the surface. In addition, the SEM image (Fig. 8d) exhibits clearly the formation of Ag nanoparticles with a diameter ranged from 200 to 20 nm. The presence of the Ag nanoparticles was also confirmed using surface plasmon resonance as shown in the inset in Fig. 8d. The spectrum shows the presence of plasmon band at 572 nm confirming the formation of Ag nanoparticles. Furthermore, for ITO cathodic polarization at −2.6 V followed by immersion in AgClO_4_ solution, the SEM image (Fig. S3) shows clearly the presence of ITO islands and Ag nanoparticles. This observation indicates that even after the damage of the surface the activity of the reduced ITO is preserved at microscopic scale and able to reduce Ag cation.

In summary, this work demonstrates the first example of the cathodic modification of the ITO electrode using mild electrochemical reducing conditions. This puzzling process was performed using alkali metal and redox active electrolyte. The cathodic polarization of ITO conduces to a morphological and chemical composition change of the surface. The XPS, X-ray diffraction and electrochemical investigations demonstrate clearly the intercalation of the electrolyte cation into the ITO materials and the electrochemical reversibility of the process. Furthermore, the reducing power of the electrochemically treated ITO was successfully used to induce electro-less reduction of Ag^+^. Interestingly, a multifunctional active and transparent ITO surface was generated bearing redox active molecule inside the material and Ag nanoparticles onto the surface. Besides the fundamental interest of this topic, we believe that the possibility to intercalate alkali metals or redox molecules into the ITO materials will enhance the possibility to tune the specific properties of the ITO interface and their integrations in several devices for applications devoted to electronic and energy storage.

## Methods

### Chemicals

Sodium iodide (NaI), potassium chloride (KCl), silver perchlorate (AgClO_4_), anhydrous acetonitrile, (ferrocenylmethyl)trimethylammonium iodide, imidazole, methyl iodide and tetrabutylammonium tetrafluoroborate (Bu_4_NBF_4_) were supplied from Sigma-Aldrich at the highest grade available. The synthesis pathway of 1-ferrocenylmethyl-3-methylimidazolium iodide is described in Supporting Information, Fig. S1.

### Electrochemistry

Electrochemical measurements were performed using CHI 660 work station (CH Instruments, Texas, USA). Classical three electrode cell was used, an indium tin oxide (ITO, 30–50 Ω/sq) coated glass sample (supplied from SOLEMS, France) was used as working electrode, platinum wire and Ag/AgCl were used as counter and reference electrodes, respectively. Before the electrochemical measurements the solutions were deoxygenated and during the experiments the cell remains under argon atmosphere.

### Surfaces analysis

The XPS investigations were achieved using a Thermo VG Scientific ESCALAB 250 system with a monochromatic Al kα (hυ = 1486.6 eV). For the survey and the high resolution spectra the used pass energy was 100 and 40 eV, respectively. Data acquisition and processing were performed using the Avantage software. The atomic percentages were calculated using the software and considering photoemission cross sections, analyzer transmission and the variation of the electron mean free paths. Scanning electron microscope images, SEM, were recorded using Zeiss Supra 40 instrument. Absorption measurements were performed using spectrophotometer Ocean Optics (HR 4000) coupled with a fiber-optic system. X-ray diffraction was performed using an Empyrean diffractometer from Panalytical equipped with a multichannel PIXcel 3D detector and a filtered-copper X-ray source (1.5418 Å) in the 10–70° 2θ range.

## Additional Information

**How to cite this article**: Bouden, S. *et al*. Multifunctional Indium Tin Oxide Electrode Generated by Unusual Surface Modification. *Sci. Rep.* **6**, 36708; doi: 10.1038/srep36708 (2016).

**Publisher’s note:** Springer Nature remains neutral with regard to jurisdictional claims in published maps and institutional affiliations.

## Supplementary Material

Supplementary Information

## Figures and Tables

**Table 1:** XPS Atomic Percentages for Blank and Reduced ITO at −2 V in ACN Containing 0.1 M NaI at Different Charges Densities.

Charge (C.cm^−2^)	0	−0.4	−1.6	−2.4	−4.7
In 3d_5/2_ (At%)	29	20	9	8.5	4
Sn 3d_5/2_ (At%)	3.4	2.7	0.4	0.9	0.3
Na 1s (At%)	0	1.6	12.7	15.8	16.6
I 3d_5/2_ (At%)	0	0.1	0.9	1.1	0.7
