# Supplementary material for: Multifunctional Indium Tin Oxide Electrode Generated by Unusual Surface Modification
Source: Sci Rep. 2016 Nov 18;6:36708. doi: 10.1038/srep36708 (PMC5114566; doi:10.1038/srep36708)
Supplement: Supplementary Information [file srep36708-s1.pdf]

Multifunctional Indium Tin Oxide Electrode Generated by Unusual Surface Modification

Sarra Bouden,[1] Antoine Dahi,[1] Fanny, Hauquier,[2] Hyacinthe Randriamahazaka,[1] Jalal Ghilane[1*]

[1]Nano-Electro-Chemistry group, Univ Paris Diderot, Sorbonne Paris Cité, ITODYS, UMR 7086 CNRS, 15 rue Jean-Antoine de Baïf, 75205 Paris, France.

[2] LICSEN, NIMBE, CEA, CNRS, Université Paris-Saclay, CEA Saclay 91191 Gif-sur-Yvette Cedex, France - CNAM, Department of Chemistry and Health & Life Sciences, 292 Rue Saint-Martin, 75003 Paris, France.

Email : jalal.ghilane@univ-paris-diderot.fr

Experimental details.

Synthesis of 1-ferrocenylmethyl-3-methylimidazolium iodide.

The synthesis pathway of *1-ferrocenylmethyl-3-methylimidazolium iodide* is summarized in Fig. S1.

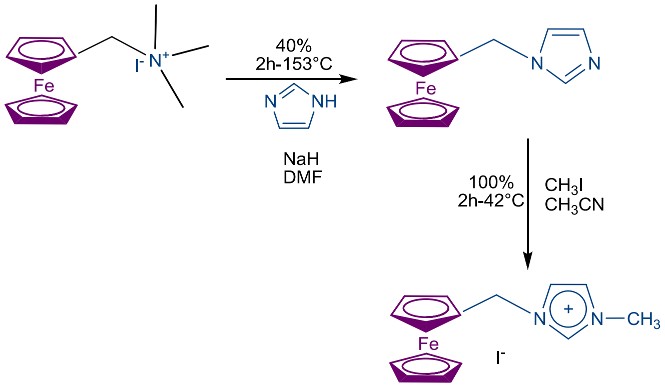

Figure S1. Synthesis pathway of 1-ferrocenylmethyl-3-methylimidazolium iodide.

Briefly, (ferrocenylmethyl)trimethylammonium iodide (1 equivalent) and imidazole (1.22 equivalents) were dissolved in 200 mL of DMF. The solution was refluxed for 2 h and a black solid phase was separated by filtration. Next, the synthetized 1-(ferrocenylmethyl) imidazole (1 equivalent) was dissolved in acetonitrile (20 mL), and then methyl-iodide (3 equivalents) was added drop-wisely before refluxing for 2 h at 42°C. After that, solvent and methyliodide were removed by rotavapor and an orange solid was obtained. The product was then placed under vacuum for 24 hrs.

^{1}H NMR (400 MHz, DMSO) δ: 3.82 (s, 3H), 4.23-4.24 (m, 7H), 4.44 (t, J = 1.6 Hz, 2H), 5.16 (s, 2H), 7.65 (s, 1H), 7.73 (s, 1H), 9.07 (s, 1H).

Figures for Supporting Information.

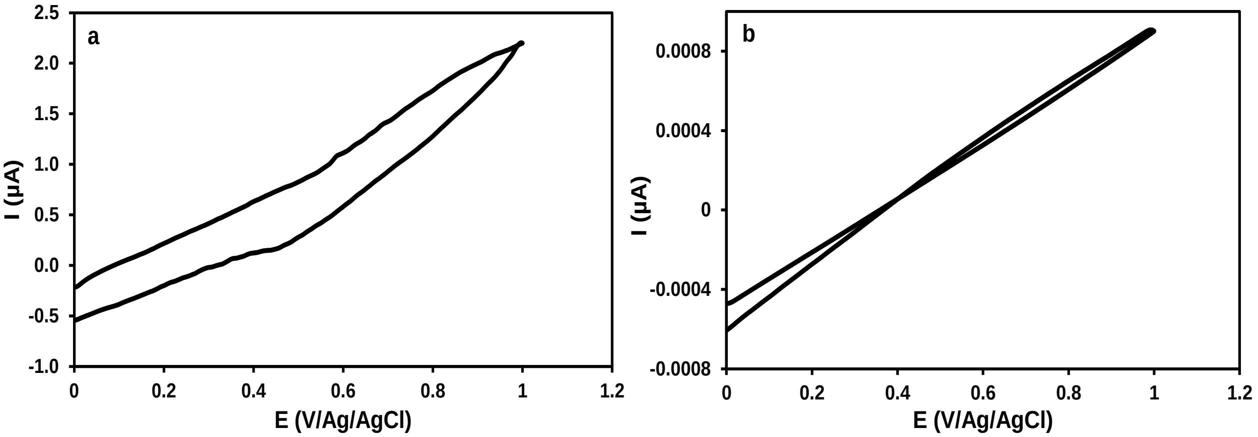

Figure S2. CV's of reduced ITO electrode in acetonitrile solution containing 0.1 M Bu_4NBF_4, (a) after cathodic polarization at -2.4 V during 100 s and (b) after cathodic polarization at -2.6 V during 100 s. scan rate 0.1 $V.s^{-1}$.

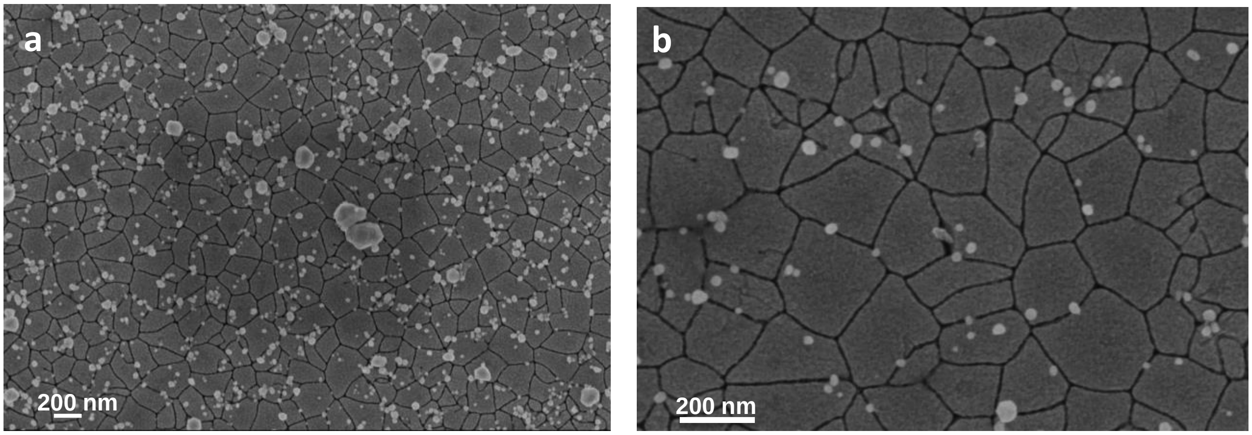

Figure S3. SEM image of ITO sample after cathodic treatment at -2.6 V during 100 s followed by immersion in aqueous solution containing 0.1 M $AgClO_4$.
